# Supplementary material for: Human IL-34 Deficiency Primes Microglia Toward Alzheimer’s Disease-Associated States
Source: medRxiv. 2026 Jun 25:2026.02.21.26346696. Preprint. [Version 2] doi: 10.64898/2026.02.21.26346696 (PMC13321156; doi:10.64898/2026.02.21.26346696)

medRxiv preprint doi: https://doi.org/10.64898/2026.02.21.26346696; this version posted June 25, 2026. The copyright holder has placed this preprint (which was not certified by peer review) in the Public Domain. It is no longer restricted by copyright. Anyone can legally share, reuse, remix, or adapt this material for any purpose without crediting the original authors.

Supplementary Figure Legend:

Supplementary Figure 1. The IL-34-Y213X variant is associated with reduced IL-34 levels in cerebrospinal fluid. *Box plots showing CSF IL-34 levels in individuals stratified by IL-34 genotype (Y213/Y213, Y213/X, and X213/X213), measured across three independent experiments using two orthogonal proteomic platforms: two SomaScan assays (left and middle panels) and one Olink immunoassay (right panel). Across all platforms, carriers of the IL-34-Y213X variant show a marked reduction in CSF IL-34 levels. p values indicate statistical significance of genotype effects.*

Supplementary Figure 2. Genome-wide associations of IL-34 locus variants and related phenotypes. *(A) Heatmap of genome-wide significant associations for IL-34 locus variants extracted from the GWAS Catalog. Multiple traits reach statistical significance, with strongest signals observed for hippocampal volume, cortical thickness, and brain MRI–derived traits, alongside additional associations with Alzheimer's disease risk, cognitive performance, mathematical ability, and systemic traits such as APOL1 protein levels. (B) System-level distribution of traits associated with IL-34 locus variants. Pleiotropic effects are evident across multiple biological domains, with a clear predominance in brain- and eye-related phenotypes, but also extending to metabolism, immune/inflammatory traits, and systemic physiology. (C) Regional Manhattan plot of the IL-34 locus (chr16:66.9–73.0 Mb, GRCh38).*

Supplementary Figure 3. STRING protein–protein interaction network from protein level data and cluster-specific pathway enrichment. *STRING PPI network is constructed from proteins with altered levels. Each node represents a protein and edges denote predicted interactions. Node colors indicate cluster membership, with the largest modules corresponding to Cluster 1 (red): Axon guidance, and Cluster 2 (light-orange): Positive regulation of humoral immune response. Smaller modules include galactose metabolism, collagen chain trimerization, and others. Network statistics (inset) confirm significant enrichment over random expectation.*

Supplementary Figure 4. Cross-species comparison of human genetic IL-34 deficiency and IL-34KO mouse microglial transcriptomics. *(A) Schematic overview of the cross-species integration strategy comparing the human CSF SomaScan*

medRxiv preprint doi: https://doi.org/10.64898/2026.02.21.26346696; this version posted June 25, 2026. The copyright holder has placed this preprint (which was not certified by peer review) in the Public Domain. It is no longer restricted by copyright. Anyone can legally share, reuse, remix, or adapt this material for any purpose without crediting the original authors.

differential signature from IL-34 Y213X homozygotes versus matched wild-type controls with the mouse IL-34KO versus wild-type microglial transcriptome. (B) Gene set enrichment analysis showing the enrichment trend of human proteins increased in IL-34-deficient individuals within the ranked mouse IL-34KO microglial transcriptome. Positive ranks indicate genes increased in IL-34KO microglia. (C) Rank-level comparison across all mapped human–mouse orthologs using signed significance metrics for the human proteomic and mouse transcriptomic contrasts. Positive human ranks indicate increased protein abundance in IL-34 Y213X homozygotes, and positive mouse ranks indicate increased expression in IL-34KO microglia.

Supplementary Figure 5. Structural modeling of the IL-34 Y213X truncation. *AlphaFold-predicted structure of IL-34 indicating the position of the Y213 residue and the resulting Y213X truncation. The truncation localizes to the unstructured C-terminal region, distal from the folded core of the protein, suggesting that the overall globular structure is preserved and that major disruption of somamer- or antibody-binding epitopes is unlikely.*

medRxiv preprint doi: https://doi.org/10.64898/2026.02.21.26346696; this version posted June 25, 2026. The copyright holder has placed
preprint (which was not certified by peer review) in the Public Domain. It is no longer restricted by copyright. Anyone can legally share, re
remix, or adapt this material for any purpose without crediting the original authors.

Supplementary Figure 1

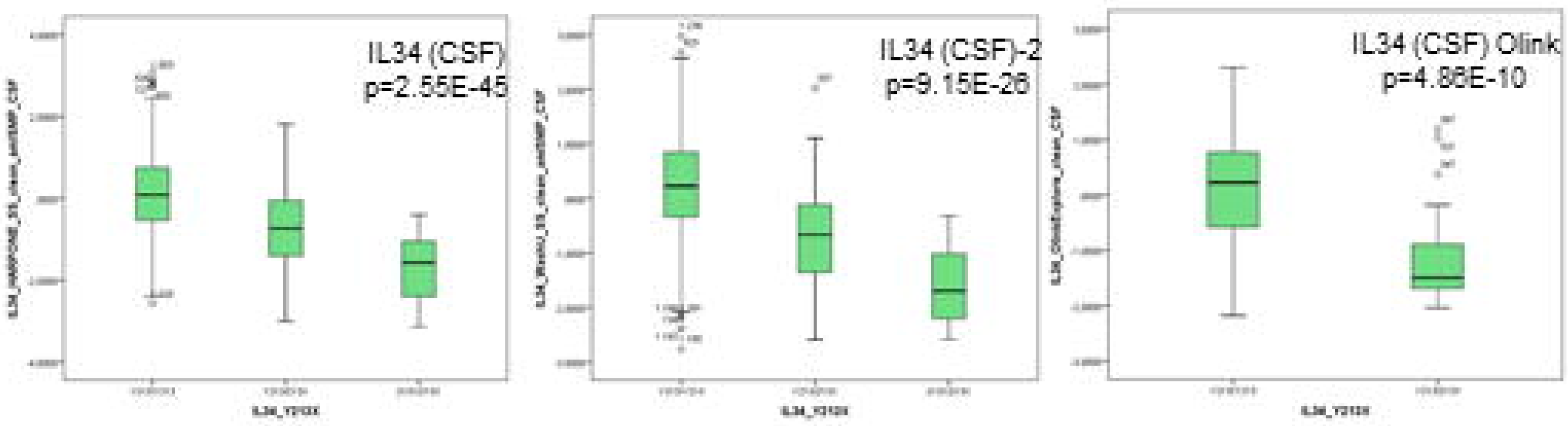

medRxiv preprint doi: https://doi.org/10.64898/2026.02.21.26346696; this version posted June 25, 2026. The copyright holder has placed this preprint (which was not certified by peer review) in the Public Domain. It is no longer restricted by copyright. Anyone can legally share, reuse, remix, or adapt this material for any purpose without crediting the original authors.

Supplementary Figure 2

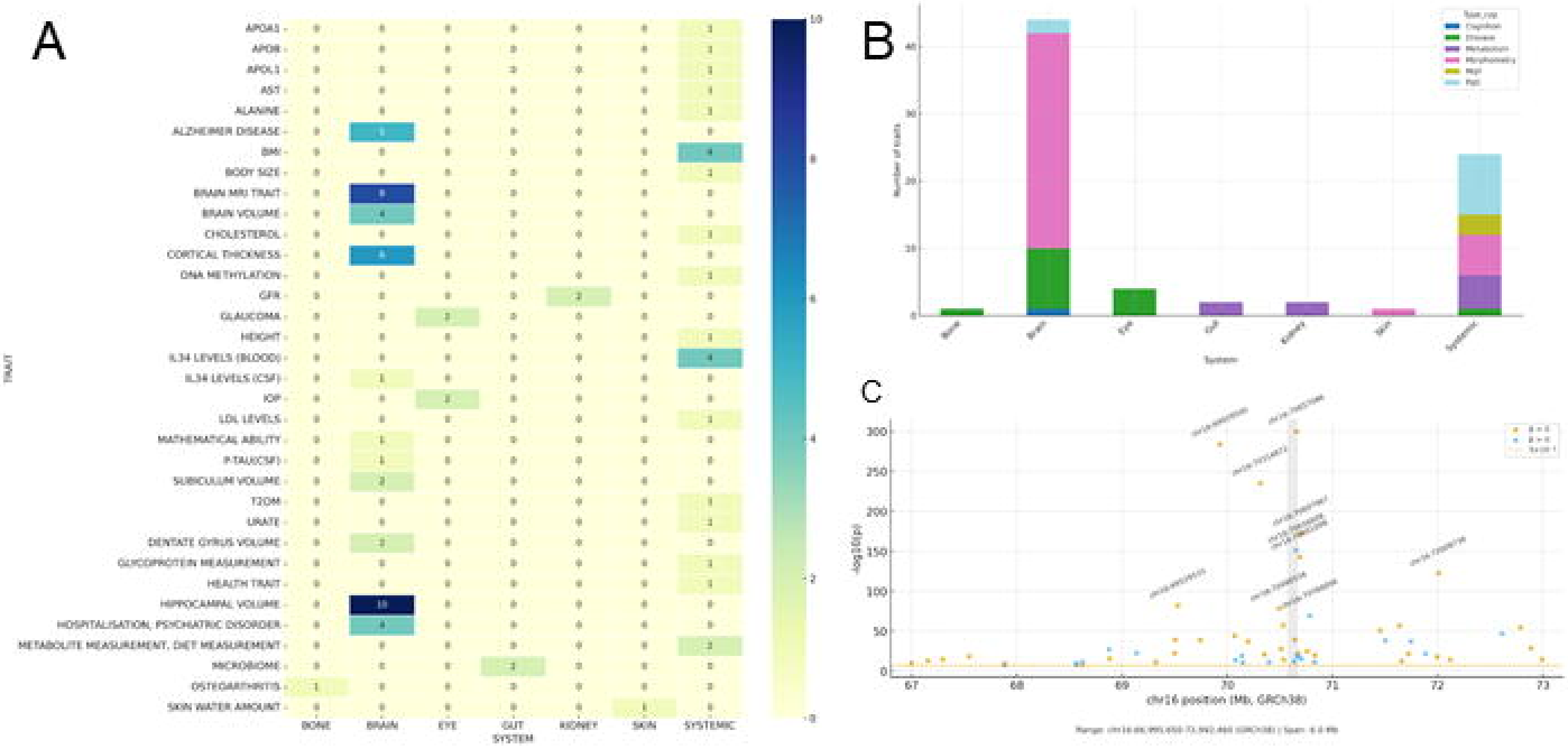

medRxiv preprint doi: https://doi.org/10.64898/2026.02.21.26346696; this version posted June 25, 2026. The copyright holder has placed
preprint (which was not certified by peer review) in the Public Domain. It is no longer restricted by copyright. Anyone can legally share, re
remix, or adapt this material for any purpose without crediting the original authors.

Supplementary Figure 3

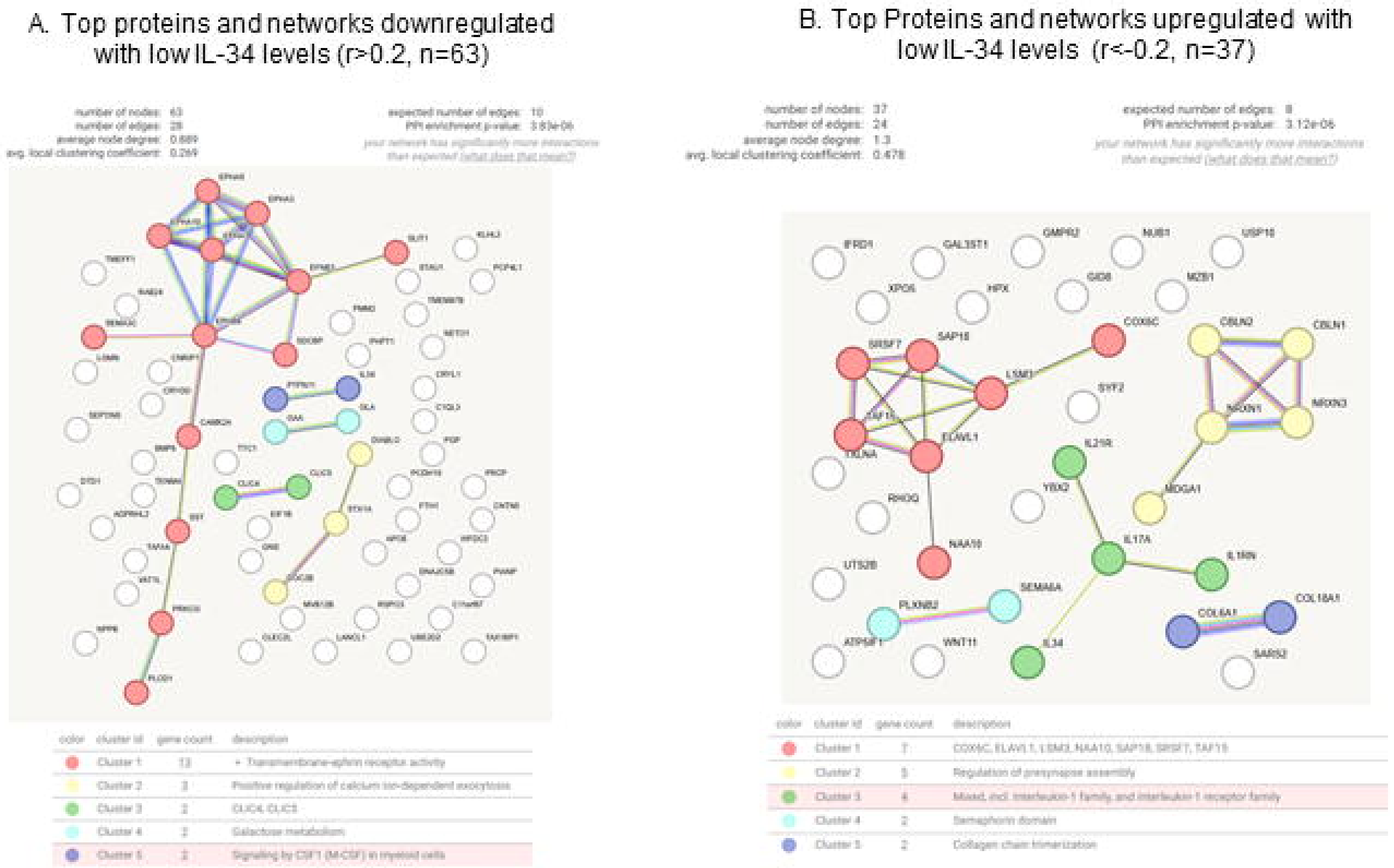

medRxiv preprint doi: https://doi.org/10.64898/2026.02.21.26346696; this version posted June 25, 2026. The copyright holder has placed
preprint (which was not certified by peer review) in the Public Domain. It is no longer restricted by copyright. Anyone can legally share, re
remix, or adapt this material for any purpose without crediting the original authors.

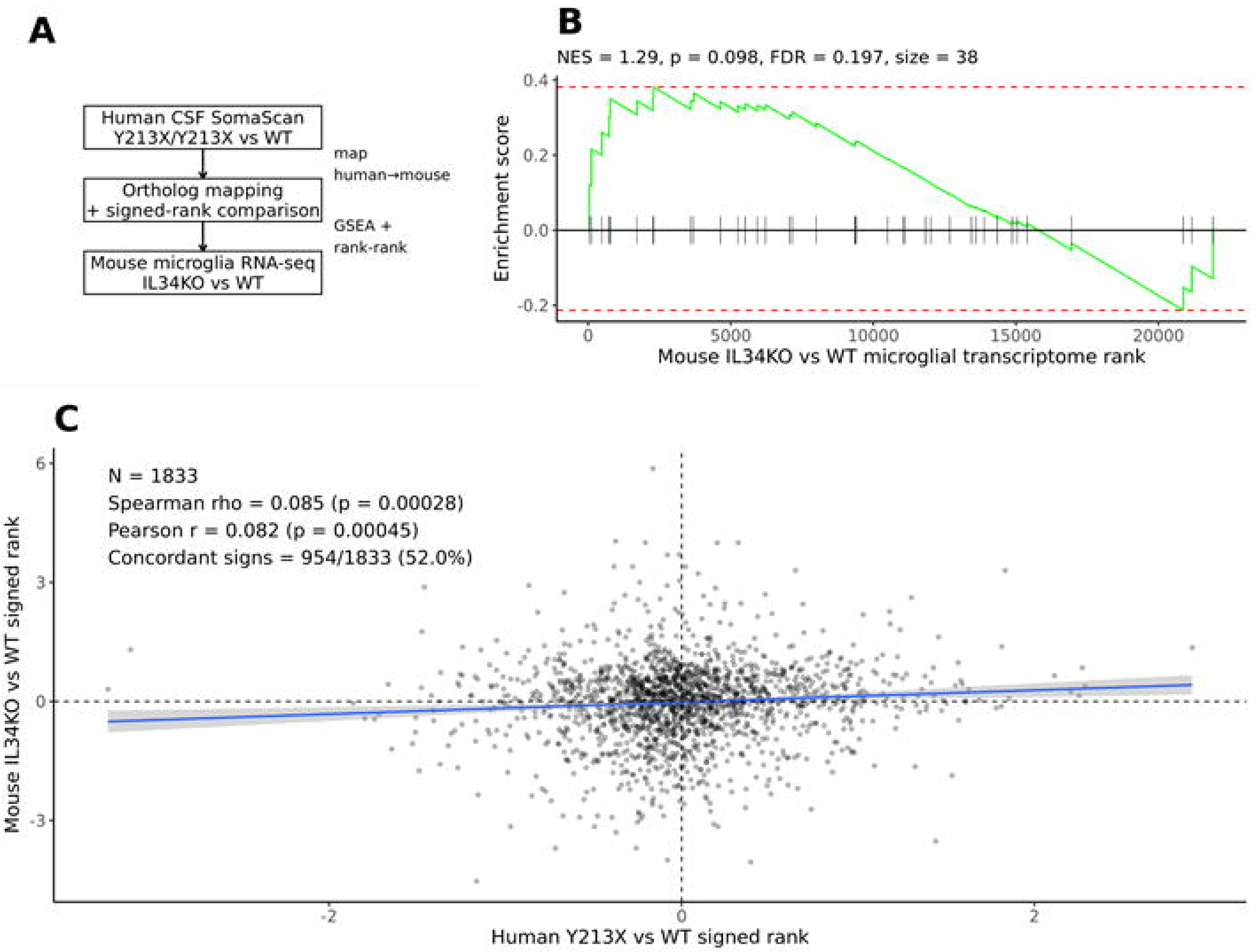

Supplementary Figure 4

medRxiv preprint doi: https://doi.org/10.64898/2026.02.21.26346696; this version posted June 25, 2026. The copyright holder has placed
preprint (which was not certified by peer review) in the Public Domain. It is no longer restricted by copyright. Anyone can legally share, re
remix, or adapt this material for any purpose without crediting the original authors.

Supplementary Figure 5

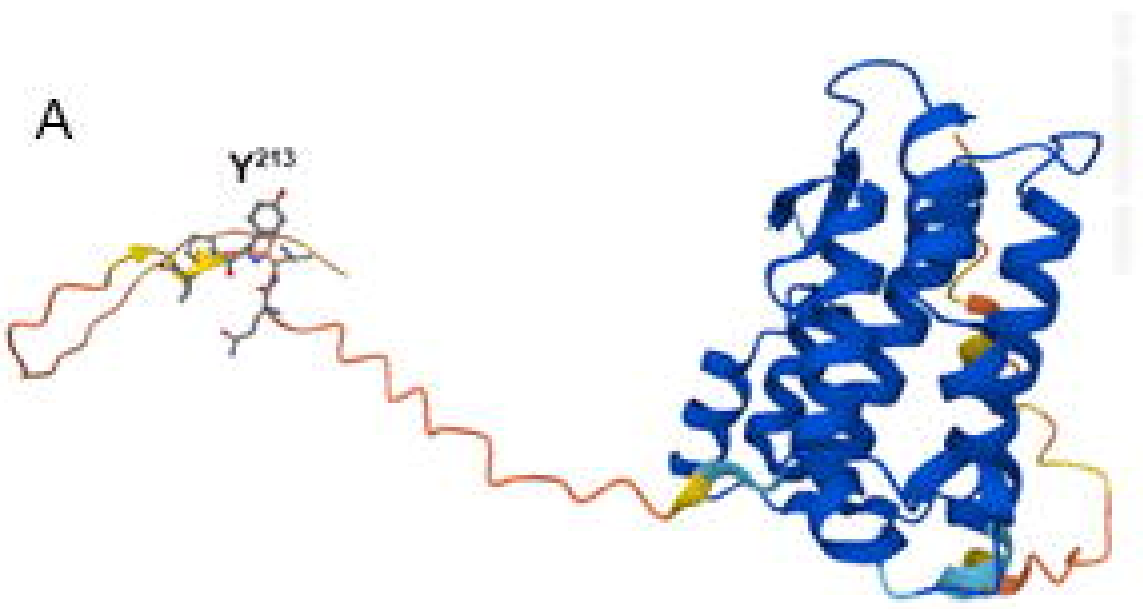

Supplement: Supplementary file 1 [file NIHPP2026.02.21.26346696v2-supplement-4.pdf]
